# Application Method Determines Effects of *Beauveria bassiana* on *Eucalyptus grandis* Growth and Leaf-Cutting Ant Foraging

**DOI:** 10.3390/insects17020134

**Published:** 2026-01-24

**Authors:** Raymyson Rhuryo de Sousa Queiroz, Thais Berçot Pontes Teodoro, Aline Teixeira Carolino, Ricardo de Oliveira Barbosa Bitencourt, Richard Ian Samuels

**Affiliations:** Laboratório de Entomologia e Fitopatologia, Universidade Estadual do Norte Fluminense Darcy Ribeiro, Campos dos Goytacazes, Rio de Janeiro 28013-602, Brazil; queirozraymyson@gmail.com (R.R.d.S.Q.); thaisbercot1@gmail.com (T.B.P.T.); carolinoat@gmail.com (A.T.C.); ricoliver@gmail.com (R.d.O.B.B.)

**Keywords:** *Acromyrmex subterraneus subterraneus*, insect behavior, endophyte–plant interaction, entomopathogenic fungi

## Abstract

*Beauveria bassiana* can colonize plants, acting against insect pests and promoting plant growth. This study evaluated how different fungal inoculation methods affected *Eucalyptus grandis* growth and the foraging behavior of ants. An isolate (LPP 139) was identified as *B. bassiana* based on ITS sequences. Seedlings were submitted to three inoculation methods using fungal suspensions at 1 × 10^8^ conidia mL^−1^: (1) soil drenching at sowing (SD), (2) soil drenching 20 days after sowing (20SD), and (3) foliar spraying 20 days after sowing (20F) when compared to controls. SD treatment enhanced plant height (mean 25 cm with a 31.6% increase compared to the controls; *p* = 0.0353) and shoot fresh weight (mean 1.5 g, a 50% increase; *p* = 0.0154), while 20SD increased leaf number (141.4% increase; *p* = 0.0419). The 20F treatment increased leaf number (287.9% compared to the controls; *p* = 0.0006), shoot weight (mean fresh weight 1.5 g, a 50% increase; *p* = 0.0213 and mean dry weight 0.7 g, a 75% increase; *p* = 0.0236), and reduced leaf-cutting ant foraging (mean 26 cm^2^, a reduction of 53.6%; *p* = 0.0134). These findings highlight the dual action of *B. bassiana*, promoting plant growth and reducing the activity of ants.

## Simple Summary

Leaf-cutting ants pose a severe threat to South American eucalyptus plantations, leading to significant economic losses. Traditional management relies on synthetic chemical baits, which are notorious for their environmental toxicity and long-term persistence in the soil. Consequently, alternative control strategies are urgently needed. While entomopathogenic fungi can be applied directly to ants or their nests, they also offer a novel approach to plant protection through plant tissue colonization. These fungi can act as endophytes, living within the plant, to provide protection and stimulate growth. However, the effectiveness of this protection and growth stimulation often depends on the inoculation method used. In this study, we tested three inoculation methods. The most promising results occurred when eucalyptus leaves were sprayed with fungal suspensions 20 days after planting. This treatment both stimulated plant development and significantly reduced leaf-cutting ant foraging activity. This research provides a foundation for reducing our reliance on chemical pesticides, offering a greener solution that benefits both eucalyptus growers and the broader ecosystem.

## 1. Introduction

*Eucalyptus grandis* Hill ex Maiden, commonly known as flooded gum or rose gum, is a tree species native to Australia [1], widely cultivated in tropical and subtropical regions due to its rapid growth, high productivity, and broad adaptation to different conditions [2,3]. *Eucalyptus* plantations represents a major component of Brazil’s forestry sector, supplying raw materials for construction, furniture, energy, and the pulp for paper industries [4]. As global demand for wood and wood-based products continues to rise, particularly with woodchip demand projected to grow around 40% by 2030 [5], the economic relevance of *Eucalyptus* plantations is expected to increase further. However, expanding production faces several challenges, including climate change [6], plant disease [7] and insect damage [8].

Among the most destructive pests in these forestry systems are leaf-cutting ants (*Acromyrmex* spp., *Atta* spp., Hymenoptera: Formicidae), which defoliate plants at all developmental stages, reducing biomass accumulation and overall productivity [9,10]. These ants harvest fresh leaves to cultivate a symbiotic fungus (*Leucoagaricus gongylophorus*) as their primary food source, making them highly efficient herbivores and one of the main obstacles to production in South America [11,12].

Chemical control, primarily utilizing sulfluramid-based baits, remains the most widely adopted strategy for managing leaf-cutting ants. This preference is attributed to its high efficacy, achieved through colony-level transmission once the insecticide is carried into the nest [13]. However, the synthesis of sulfluramid requires perfluorooctane sulfonyl fluoride (PFOSF), a known precursor of Persistent Organic Pollutants (POPs), and sulfluramid itself degrades in the environment, resulting in the formation of perfluorooctanesulfonic acid (PFOS). These compounds raise significant environmental concerns due to their persistence, bioaccumulation [14], toxicity to aquatic organisms [15] and mammals [16]. Consequently, global regulatory restrictions have been imposed on the use and production of sulfluramid [17].

In this context, biological control, particularly through the use of entomopathogenic and antagonistic fungi, has emerged as a promising and sustainable alternative for managing leaf-cutting ants and other agricultural pests. This strategy offers reduced environmental impact, low non-target toxicity, and potential for host specificity [18,19,20,21,22]. Compared to chemical insecticides, fungal-based control agents are considered environmentally safer because they cause less damage to the environment and non-target insects [23]. The entomopathogenic fungi (EPF) *Beauveria* spp., *Metarhizium* spp., *Hirsutella* spp., *Lecanicillium* spp., and *Paecilomyces* are the most widely studied genera for controlling various agricultural insect pests [23,24], including leaf-cutting ants [25,26].

Beyond their role as entomopathogens, several strains of these fungi are capable of colonizing plant tissues endophytically, providing dual benefits by promoting plant growth, enhancing resistance to biotic stress, and deterring herbivory [27,28,29,30]. Endophytic colonization typically occurs through the adhesion of fungal conidia to the plant surface, followed by germination and penetration via natural openings such as stomata, lenticels, root cracks, and germinating radicles, or through direct enzymatic degradation of the cell wall [31,32]. The promotion of plant development by endophytic *Beauveria bassiana* is associated with multiple physiological mechanisms, including improved nutrient uptake [33], modulation of phytohormone levels such as auxins and gibberellins [34], and stimulation of antioxidant enzyme activity [35].

As far as we know, there is little information about the cross-link between the endophytic activity of *B. bassiana* in *Eucalyptus grandis* and its impact on the foraging behavior of leaf-cutting ants in this plant species. Here, we studied the endophytic activity of *B. bassiana* in *E. grandis* via multiple inoculation methods, as well as the influence of treating plants with this fungus on plant growth parameters. Furthermore, the protective effect of endophytes on *E. grandis* was assessed when considering the foraging behavior of the leaf-cutting ant *Acromyrmex subterraneus subterraneus*.

## 2. Materials and Methods

### 2.1. Collection and Maintenance of Leaf-Cutting Ant Colonies

Six *Acromyrmex subterraneus subterraneus* nests were collected in the field from Bom Jardim city, Rio de Janeiro State, Brazil (22°09′07″ S and 42°25′10″ W). The nests were carefully excavated with the queens, workers, and their symbiotic fungus *L. gongylophorus*. The colonies were placed in 2L plastic containers and transported to the Myrmecology Unit of the Universidade Estadual do Norte Fluminense Darcy Ribeiro (UENF). The *Acromyrmex* species was identified according to [36]. The colonies were kept under controlled condition [12:12 h light:dark cycle, 26 ± 2 °C, and 70% ± 5% relative humidity (RH)]. The fungus *L. gongylophorus* was cultivated by the ants in plastic containers placed in trays and the sides of the trays were coated with talcum powder to prevent insect escape. Fresh *Acalypha wilkesiana* leaves were placed daily in the foraging arenas.

### 2.2. Fungus: Isolation and Molecular Characterization

*Beauveria* sp. LPP 139 was originally found infecting an adult coffee berry borer *Hypothenemus hampei* (Ferrari) (Coleoptera: Curculionidae, Scolytinae). To obtain a pure isolate of this fungus, insect-derived conidia derived from a moribund insect were collected directly with a sterile inoculation loop and transferred to Potato Dextrose Agar (PDA: KASVI, Curitiba, Brazil) medium supplemented with 0.05% chloramphenicol (Sigma, St. Louis, MO, USA) and incubated under controlled conditions (25 ± 2 °C, RH = 70%). After 15 days, the fungus was purified using a monosporic culture technique as described by [37].

For molecular characterization, approximately 50 mg of conidia were collected from PDA cultures and genomic DNA was extracted using the Quick-DNA™ Fungal/Bacterial Miniprep Kit (Zymo Research, Irvine, CA, USA) following the manufacturer’s instructions. DNA concentration and purity were assessed using a NanoDrop 2000 spectrophotometer (Thermo Scientific, Waltham, MA, USA). The internal transcribed spacer regions ITS-1 (TCCGTAGGTGAACCTGCGG) and ITS-4 (TCCTCCGCTTATTGATATGC) were employed for PCR amplification to facilitate fungal identification [38]. PCR reactions were prepared with 4.0 µL of dNTPs (1.25 mM), 5.0 µL of 5× buffer, 2.0 µL of MgCl_2_ (25 mM), 0.5 µL of Taq polymerase (5 U/µL), 1.0 µL of each primer (10 µM), 10.5 µL of ultrapure water, and 2.0 µL of DNA template (10 ng). Thermal cycling conditions consisted of an initial denaturation at 96 °C for 3 min, followed by 35 cycles of denaturation at 94 °C for 45 s, annealing at 55 °C for 60 s, and extension at 72 °C for 120 s, with a final extension at 72 °C for 10 min. PCR products were separated on 1% agarose gels, stained with GelRed (Biotium, Fremont, CA, USA), and visualized under UV illumination. Amplified fragments were purified using ExoSAP-IT PCR Product Cleanup Reagent (Applied Biosystems, Waltham, MA, USA) according to the manufacturer’s instructions, and subsequently sequenced by Sanger sequencing at ACTGene Análises Moleculares (Nova Alvorada, RS, Brazil).

The sequences obtained in this study were compared against 13 ITS sequences of various *Beauveria* species retrieved from the GenBank database (Table A1). *Nakaseomyces glabratus* (H. W. Anderson) Sugita & Takashima (Saccharomycetales: Saccharomycetaceae) was designated as the outgroup (Table A1). Chromatogram editing was performed using DNA Baser v.5. Sequence alignment was conducted through the Clustal W progressive method. A phylogenetic tree was generated using the maximum likelihood algorithm with nucleotide distances calculated according to the Kimura 2-parameter model. Tree robustness was evaluated via bootstrap analysis with 1000 replicates, employing the MEGA version 11 software [39].

### 2.3. Fungal Suspension

*Beauveria* sp. LPP 139 was cultivated on Petri dishes containing PDA and maintained controlled condition (25 ± 2 °C, RH = 70%). After 15 days, the conidial suspensions were prepared in sterilized distilled water containing 0.05% (*v*/*v*) Tween 80 (Quimiobrás Industrias Químicas SA, Rio de Janeiro, RJ, Brazil). For all experiments, the conidial suspension was quantified using a hemocytometer and adjusted to 1 × 10^8^ conidia/mL [40].

### 2.4. Treatment of Eucalyptus with Conidial Suspensions

Commercial *Eucalyptus grandis* seeds, LCFA 002 cultivar, obtained from Instituto de Pesquisa e Estudos Florestais (Piracicaba, Brazil), were surface-sterilized by immersion in 70% ethanol for 1 min, followed by 5% sodium hypochlorite with Tween 20. After 10 min, the seeds were washed three times with sterile distilled water [41]. For germination, the seeds were placed on moistened filter paper in Petri dishes. After six days, seedlings were transplanted into 100 cm^3^ tubes filled with commercial substrate Basaplant (Artur Nogueira, Brazil), previously autoclaved twice at 120 °C for 30 min.

Groups of *E. grandis* seeds/plants (*n* = 15 per group) were treated with the fungal suspension using three methodologies as follows: (1) soil drenching at sowing (SD); (2) soil drenching 20 days after sowing (20SD), and (3) foliar spraying 20 days after sowing (20F). For SD and 20SD, 2 mL of a conidial suspension was applied to the soil surface around the stem base of the plant. For 20F, 1.5 mL of a conidial suspension was manually sprayed onto each plant, which was the optimal volume determined to ensure adequate leaf coverage without causing runoff or drift of the suspension to the soil. Additionally, in the 20F treatment groups, the upper surface of the pots surrounding the plant stem was covered with aluminum foil prior to foliar spraying, thereby preventing contact between the soil and the fungal suspension. Following pulverization, transparent plastic bags were used to cover the plants for 24 h, in order to maintain humidity and facilitate fungal germination and colonization [42]. Plants were grown under shaded greenhouse conditions (50% shading) and irrigated as needed.

### 2.5. Confirmation of the Endophytic Colonization in Eucalyptus

After 90 days, five plants per group were randomly selected to assess endophytic colonization. Two g of stems, leaves, and roots were surface-washed, macerated, and serially diluted (up to 10^−2^) as described by [43] with modifications. Aliquots with 800 μL from each dilution were plated on PDA and incubated at 27 °C under a 12:12 h photoperiod. The growth of microorganisms was monitored daily, and re-isolations were performed to confirm the presence of *B. bassiana* following the protocol described by [44] with modification. Here, fungal growth was observed as discrete, circular colonies resembling colony forming units.

### 2.6. Biometric Parameters of Eucalyptus Treated with Conidial Suspension

After 90 days, biometric parameters were measured on *Eucalyptus* plants (N = 5 per group) exposed to the fungus. The number of leaves, leaf area (cm^2^), plant height (cm), stem diameter (mm), fresh and dry weight of shoots and roots (g) were measured. Fresh weights of the shoots and roots were taken immediately after the plants were collected from the greenhouse. Dry weights were determined after drying the plant material in a forced-air oven (Ethik 400D, Ethik Technology, Vargem Grande Paulista, SP, Brazil) at 70 °C for 24 h. The weight of the above-ground parts of the plant (i.e., leaves, stems) and roots were separately measured using an analytical balance (BEL^®^ M214A, BEL Engineering, Monza, Italy). In addition, leaf area was measured using a leaf area meter (LI-3100C LI-COR Biosciences, Lincoln, NE, USA).

### 2.7. Foraging Behavior Assessment

A foraging bioassay was conducted to evaluate the acceptance of treated plants by *A. subterraneus subterraneus*, according to [45]. The experiment followed a completely randomized design with four treatments (SD, 20SD, 20F and CTL), performed with three replicates, and conducted twice at different times, using a total of six colonies.

The experimental setup consisted of two interconnected arenas (60 cm length × 40 cm width × 18 cm height). One arena was used for foraging activity, where *E. grandis* seedlings were offered, while the second arena contained 4 L plastic containers with the ant colony and their symbiotic fungus garden. One seedling from each treatment was placed equidistantly within the foraging arena, allowing simultaneous choice and exposure to all treatments.

To ensure uniform foraging behavior, ants were deprived of leaves for 24 h prior to the experiment. The experiment was terminated once the ants had defoliated the first plant entirely, regardless of treatment, and this event was adopted as the endpoint criterion to ensure comparable foraging exposure among replicates. Leaf area was measured before and after exposure to the ants using an area meter (LI-3100C; LI-COR Biosciences, Lincoln, NE, USA).

### 2.8. Statistical Analysis

**Figure 1:**
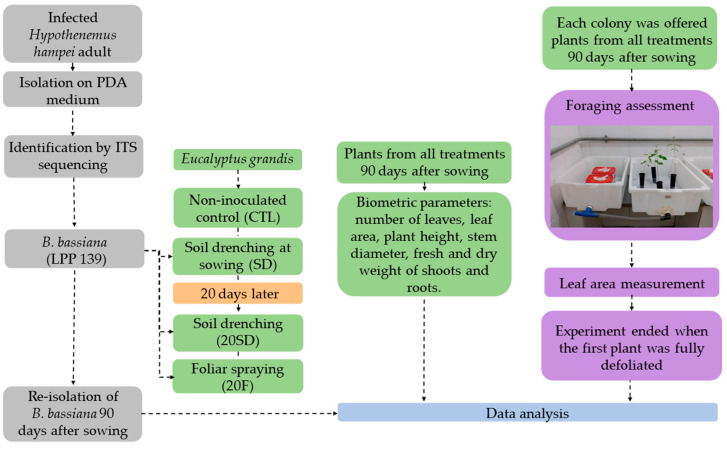
Schematic representation of the experimental workflow, summarizing all methodological steps from *Beauveria bassiana* isolation and identification to inoculation treatments, plant growth assessment, ant foraging bioassays, and statistical analysis.

All data were submitted to Shapiro–Wilk normality test. Parametric data were subjected to one-way ANOVA followed by Tukey’s multiple comparisons test (*p* ≤ 0.05), and non-parametric data were submitted to the Kruskal–Wallis test followed by Dunn’s multiple comparisons test (*p* ≤ 0.05). All statistical analyses and graphical production were performed using GraphPad Prism 8.0.2. The complete experimental workflow, illustrating all methodological steps described above, is presented in Figure 1.

## 3. Results

### 3.1. Beauveria Isolate Identification

**Figure 2:**
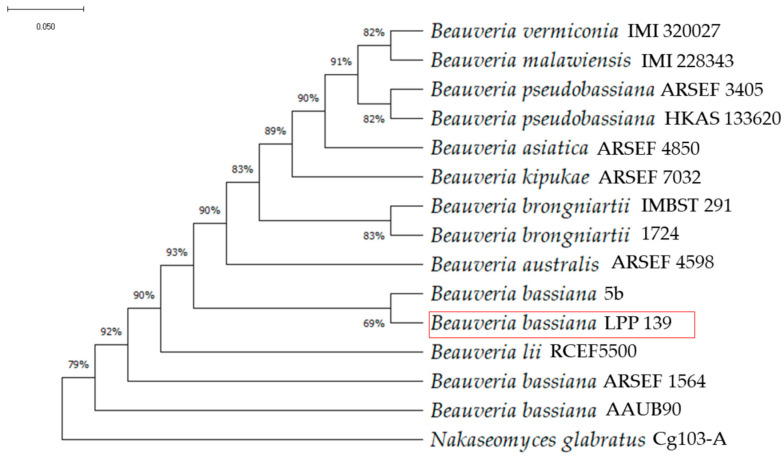
Phylogenetic tree of *Beauveria bassiana* isolate LPP 139 based on ITS rDNA sequences constructed using the maximum likelihood method. Isolate LPP 139 is highlighted within a red rectangle.

The ITS sequence obtained in this study was 492 bp in length, and a BLASTn search analysis revealed the highest nucleotide identity with *Beauveria bassiana* sequences, ranging from 91.98% to 99.22% identity and query coverage between 33% and 97%. The ITS sequence was deposited in GenBank under accession number PX591195. Also, a Bayesian phylogenetic analysis was performed, and the isolate grouped with *B. bassiana* reference sequences, confirming its taxonomic identity as *B. bassiana* (Figure 2).

### 3.2. Assessment of Endophytic Colonization

No fungal growth was observed from the aliquots of any of the evaluated samples obtained from plants at 90 days. Therefore, it was not possible to confirm systemic endophytic colonization using the currently applied technique.

### 3.3. Plant Biometric Parameters

**Figure 3:**
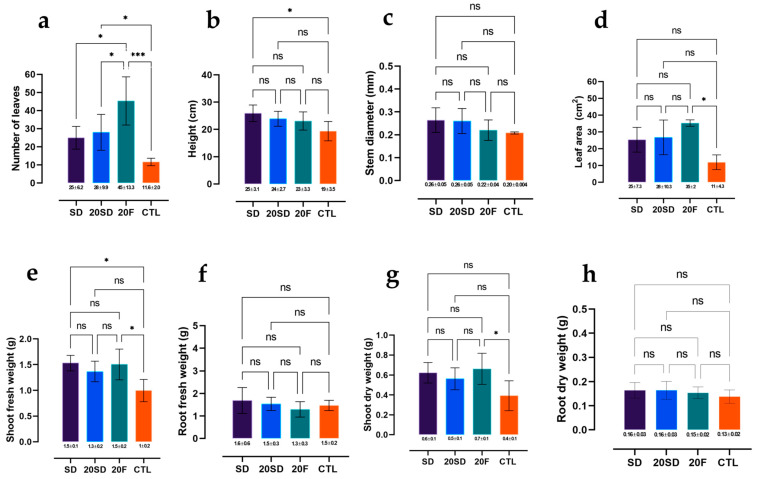
Effects of *Beauveria bassiana* inoculation on *Eucalyptus grandis* growth parameters. Graphs representing: (**a**) Number of leaves; (**b**) Height (cm); (**c**) Stem diameter (mm); (**d**) Leaf area (cm^2^); (**e**) Shoot fresh weight (g); (**f**) Root fresh weight (g); (**g**) Shoot dry weight (g), and (**h**) Root dry weight (g). Treatments are defined as: SD (Soil drenching at sowing); 20SD (Soil drenching 20 days after sowing); 20F (Foliar spraying 20 days after sowing); and CTL (Control). Bars represent standard deviation (±) of the mean. Asterisks indicated statistically significant differences (* *p* < 0.05; *** *p* < 0.001) according to Tukey’s test. ns = no significant difference between treatments.

Regarding the number of leaves (Figure 3a), comparing the groups SD vs. 20SD vs. 20F, the results were statistically different (*p* < 0.05) compared to each other. However, only SD had a similar result (*p* > 0.05) for leaf number compared with the control group. Furthermore, 20SD and 20F showed a statistical improvement in the number of leaves compared with control group (F_4,5_ = 11.05, *p* = 0.0419 and *p* = 0.0006, respectively, Figure 3a). Plants treated with fungus applied by soil drenching (SD) were significantly taller when compared with the control group (F_4,5_ = 3.415, *p* = 0.0353, Figure 3b).

Plants exposed to *B. bassiana* by foliar spray (20F) showed significantly enhancement in the leaf area compared with the control group (*p* = 0.0377, Figure 3d). The treatments SD and 20F significantly increased the shoot fresh weight compared with the control group (F_4,5_ = 5.110, *p* = 0.0154 and *p* = 0.0213, respectively, Figure 3e). Only plants exposed to fungus by foliar spraying presented an improvement in shoot dry weight compared with the control group (F_4,5_ = 4.090, *p* = 0.0236, Figure 3g). For stem diameter (Figure 3c), root fresh and dry weight (Figure 3f and h, respectively), no statistical differences (*p* > 0.05) were observed between all groups. Regardless of the treatment method (Figure 3b–h), no statistical differences (*p* > 0.05) were observed in any biometric parameters when comparing the groups treated with fungus (i.e., SD vs. 20 SD vs. 20F).

### 3.4. Ant Foraging Behavior

**Figure 4:**
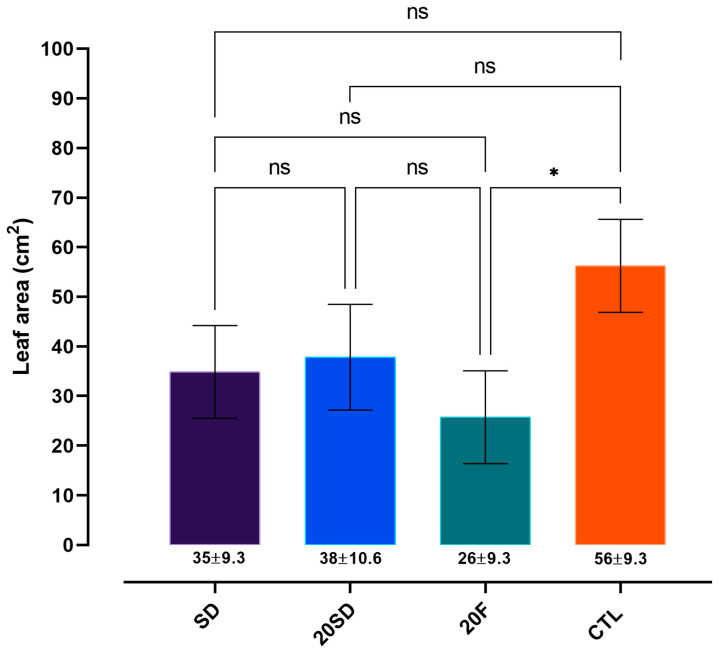
Leaf-cutting preference of *Acromyrmex subterraneus subterraneus* when offered *Beauveria bassiana*-treated *Eucalyptus grandis* plants. Leaf area (cm^2^) removed by ants. Treatments are defined as: SD (Soil drenching at sowing); 20SD (Soil drenching 20 days after sowing); 20F (foliar spraying 20 days after sowing); CTL (Control); Bars represent the standard deviations (±) of the means. Asterisks (*) indicated statistically significant differences (*p* < 0.05) when using a two-way ANOVA followed by Tukey’s post hoc test. ns = no significant difference between treatments.

Plants exposed to *B. bassiana* by foliar spray (20F) showed a significant reduction in leaf area removal by *A. subterraneus subterraneus* compared with the control group (CTL) (F_4,3_ = 5.118, *p* = 0.0134, Figure 4). In contrast, no statistical differences (*p* > 0.05) were observed among the soil drenching treatments (SD and 20SD) or between these treatments and the control group. Similarly, no significant differences (*p* > 0.05) were detected among the treatments inoculated with the fungus (SD, 20SD, and 20F) (*p* > 0.05).

## 4. Discussion

Leaf-cutting ants are considered key herbivores in Neotropical terrestrial ecosystems, exerting strong ecological pressure and causing major economic losses in diverse agricultural systems [46]. Endophytic fungi have been increasingly recognized as potential allies in plant protection, as their presence can enhance host resistance and reduce herbivory [47]. Here, it was demonstrated that the application of *B. bassiana* to *E. grandis* seedlings can both promote plant growth and reduce damage to *E. grandis* caused by leaf-cutting ants, although these outcomes are critically dependent on the method of application. Our findings provide further evidence supporting EPF as multifunctional agents in integrated crop management.

Although endophytic colonization was not confirmed through re-isolation techniques, *B. bassiana* application prompted increases in the number of leaves, plant height, leaf area, shoot fresh and dry weight (Figure 3). The success of endophyte detection is highly dependent on the chosen methodology [48] and the inability to reisolate the fungus does not necessarily indicate the absence of colonization. Fungal density [49], uneven distribution within plant tissues [50], or competition with opportunistic fungi [51], can hinder recovery by cultivation-based methods. Indeed, it has been demonstrated that colonization rates of *Beauveria* spp. can vary significantly between cultivation-dependent (e.g., re-isolation) and cultivation-independent (e.g., PCR) methods [52].

Beneficial plant responses and negative effects on insect pests following fungal inoculation were reported, even when endophytic colonization was not assessed or confirmed [53]. In a comprehensive review, summarizing various cases in which the presence of the fungus inside plant tissues was not verified, significant physiological and ecological effects were still observed [53]. For instance, inoculation of *B. bassiana* in cotton resulted in reduced performance of *Helicoverpa zea* (Lepidoptera: Noctuidae) [54], while *M. anisopliae* applied to maize produced both enhanced plant growth and decreased the survival of *Agriotes obscurus* (Coleoptera: Elateridae) [55]. These findings suggest that the influence of entomopathogenic fungi on plant–insect interactions may occur even without explicit confirmation of endophytic colonization.

The improvements observed in several growth parameters are of significant agronomic relevance for *E. grandis* cultivation [56]. Among all treatments, plants subjected to foliar inoculation with *B. bassiana* (20F) exhibited the most pronounced increases in the number of leaves, leaf area, and shoot fresh and dry weight. This superior performance compared to both soil drenching treatments and the controls suggests that the mode of inoculation influences the plant–fungus interaction. Foliar application may promote a more direct and transient contact between fungal propagules and aerial plant tissues, which could influence physiological responses such as the activation of defense-related pathways or the modulation of growth hormones [57,58]. Furthermore*, B. bassiana* can solubilize and mobilize essential nutrients such as phosphorus, iron, and zinc in the rhizosphere, thereby improving nutrient uptake efficiency [33,59]. In addition, the fungus is capable of producing phytohormone-like compounds and secondary metabolites that modulate plant hormonal balance, including auxins and gibberellins [34]. These compounds can enhance root and shoot development, increase chlorophyll content and photosynthetic capacity [60].

Here, the significant rise in the number of leaves and leaf area in the 20F treatment expands the plant’s photosynthetic surface, which may enhance carbon assimilation and contribute to biomass accumulation and wood formation [60,61]. Additionally, increased shoot, height and biomass are recognized indicators of seedling vigor [62], and more vigorous seedlings generally exhibit higher survival and establishment rates under field conditions [63].

In our results, the reduced foraging observed on plants subjected to foliar inoculation (20F) may be associated with chemical and microbial cues rather than by direct pathogenic effects. Two complementary hypotheses can be proposed. First, the foliar application itself may have acted as a repellent treatment. Ants may have detected volatile or surface cues associated with the presence of *B. bassiana*, as foliar application results in a more direct deposition of fungal conidia on the leaf surface. Consequently, the leaves may have been perceived as ‘contaminated’, deterring the ants from cutting them. Leaf-cutting ants are known to exhibit a strong hygienic behaviors and avoidance of microbial contaminants to protect their symbiotic fungus garden, and even trace amounts of entomopathogenic fungi can elicit rejection behavior [64,65,66]. Second, the initial contact between the fungus and *E. grandis* tissues may have triggered physiological adjustments in the plant, leading to the production of deterrent volatile organic compounds (VOCs) or other chemical modifications. Certain VOCs induced by fungal endophytes possess antimicrobial properties [67], which could further discourage foraging by ants seeking to minimize microbial exposure within their colonies.

Such avoidance behavior is well documented: leaf-cutting ants rely on olfactory learning to discriminate against substrates harmful to their mutualistic fungus [68,69], a process supported by long-term memory and structural remodeling in the brain [70]. Information sharing within the colony is also highly efficient, with refuse dumps acting as “information centers” where foragers associate specific odors with contaminated or unsuitable materials [71].

Altogether, these findings suggest that *B. bassiana* exerts multifaceted effects on the *E. grandis*–ant system. The method of application modulates the primary outcome: soil drenching promotes growth and vigor, whereas foliar inoculation reduces herbivory (Figure 2 and Figure 3). Future work should focus on metabolomic and volatilome analyses to identify the specific compounds mediating these effects. Understanding this functional divergence is essential for developing tailored application strategies to optimize the use of *B. bassiana* in *Eucalyptus* cultivation.

## 5. Conclusions

This study demonstrated that the method of *B. bassiana* application is a key determinant of the functional outcome in the *B. bassiana*–*E. grandis* interaction. Soil drenching at sowing (SD) significantly increased the number of leaves, shoot height, and shoot fresh weight, indicating enhanced early vigor. Foliar application at 20 days after sowing (20F) promoted even broader effects, with significant increases in leaf number, leaf area, shoot fresh and dry weights, and also resulted in a marked reduction in leaf-cutting by *A. subterraneus subterraneus*. These findings showed that *B. bassiana* can simultaneously act as a plant growth promoter and an indirect biocontrol agent, and that the application method modulates the balance between these effects. Although these results provide valuable insights into the dual functionality of *B. bassiana*, further studies are needed to elucidate the mechanisms underlying its interaction with *Eucalyptus* plants, particularly regarding colonization dynamics, endophytic detection methods, and the physiological or chemical factors mediating ant deterrence. Such advances will be crucial for optimizing fungal application strategies and consolidating *B. bassiana* as a multifunctional tool in sustainable forestry systems.

## Data Availability

Fungal sequence data is freely available at the NCBI data base under accession number PX591195. All other data will be made available on request.

## Abbreviations

The following abbreviations are used in this manuscript:

EPF	Entomopathogenic fungi
SD	Soil drenching at sowing
20SD	Soil drenching 20 days after sowing
20F	Foliar spraying 20 days after sowing
CTL	Non-inoculated control plants

## Appendix A

**Table A1:** Fungal species retrieved from the GenBank database, including accession number, query coverage, E-value, and percent identity obtained from using the NIH BLAST search tool when compared to *Beauveria bassiana* isolate LPP 139.

Specie	GenBank Accession No.	Query Cover (%)	E-Value	Percent Identity (%)
*Beauveria brongniartii* strain IMBST 291	FJ973055.1	90	0.0	97.58
*Beauveria brongniartii* strain 1724	FJ973056.1	90	0.0	97.58
*Beauveria vermiconia* strain IMI 320027	FJ973062.1	84	6 × 10^−178^	93.53
*Beauveria bassiana* strain AAUB90	MT588422.1	83	0.0	97.61
*Beauveria bassiana* ARSEF 1564	NR_111678.1	95	0.0	99.09
*Beauveria asiatica* ARSEF 4850	NR_111596.1	97	0.0	96.29
*Beauveria australis* ARSEF 4598	NR_111597.1	97	0.0	98.14
*Beauveria pseudobassiana* ARSEF 3405	NR_111598.1	97	0.0	97.22
*Beauveria kipukae* ARSEF 7032	NR_111600.1	97	0.0	97.67
*Beauveria lii* strain RCEF5500	NR_111678.1	97	0.0	97.91
*Beauveria malawiensis* IMI 228343	NR_136979.1	90	0.0	95.75
*Beauveria pseudobassiana* strain HKAS 133620	PV132939.1	89	0.0	97.26
*Beauveria bassiana* isolate 5b	GQ379914.1	87	0.0	99.22
*Nakaseomyces glabratus* voucher Cg103-A	KY981525.1	33	7 × 10^−63^	91.98
